# Urinary MicroRNA-21 for Prostate Cancer Detection Using a Silver Nanoparticle Sensor: A Promising Diagnostic Tool

**DOI:** 10.3390/bios14120599

**Published:** 2024-12-07

**Authors:** See-Tong Pang, Yueh-Er Chiou, Jasmine Lim, Yi-Chun Zhang, Wen-Zhen Zeng, Teng Aik Ong, Wen-Hui Weng

**Affiliations:** 1 Department of Urology, Chang Gung Memorial Hospital, Taoyuan City 33302, Taiwan; jacobpang@adm.cgmh.org.tw; 2 Department of Nursing, College of Medicine, Fu Jen Catholic University, New Taipei City 242, Taiwan; 031051@mail.fju.edu.tw; 3 Department of Surgery, Faculty of Medicine, Universiti Malaya, Kuala Lumpur 50603, Malaysia; jasmine.lim@um.edu.my; 4 Department of Chemical Engineering and Biotechnology, Graduate Institute of Biochemical and Biomedical Engineering, National Taipei University of Technology, Taipei City 10608, Taiwan; louis311721@gmail.com (Y.-C.Z.); t111738039@ntut.org.tw (W.-Z.Z.)

**Keywords:** biosensor, miR-21, prostate cancer

## Abstract

In this study, we detected the expression levels of miR-21 in 38 clinical urine samples, obtained from 10 patients with PCa (with each sample obtained at three time points: before surgery, 1 month after surgery, and 3 months after surgery), 3 patients with benign prostatic hypertrophy (BPH), and 5 healthy subjects (as a control group). All of the samples were examined using a silver nanoparticle-based biosensor, and the sensitivity of the biosensor was simultaneously confirmed via qRT-PCR. The results were further analyzed together with clinical data such as PSA values and cancer stages. The sensitivity of the biosensor ranged up to 100 fM, and it presented a rather high selectivity rate. Our results indicated a significantly decreased expression level of miR-21 in the patient cases at 3 months post-surgery when compared with pre-surgery levels (*p*-value < 0.001). In addition, when distinguishing the differences in the expression level of miR-21 between healthy subjects and patients with PCa or BPH, both groups showed highly significant differences (*p* = 0.008 and *p* < 0.001, respectively). The results strongly suggest that the proposed miR-21 biosensor can be used as an auxiliary screening tool for the early detection of PCa and may effectively facilitate tracking of the progression of PCa, thus enabling more accurate healthcare decision making.

## 1. Introduction

Prostate cancer (PCa) is one of the most prevalent cancers among men worldwide. Higher mortality rates are often seen in developed countries, and older age is a significant determinant of PCa. Therefore, early screening and diagnosis of PCa are crucial for patients to obtain appropriate medical options, which may significantly improve their prognosis and mortality. MicroRNA-21 (miRNA-21) has emerged as a promising biomarker for the diagnosis and prognosis of PCa due to its dysregulation in PCa cells. The epidemiology of prostate cancer (PCa) mortality reflects the complex interactions between multiple factors, with mortality rates varying widely in different regions of the world; interestingly, these rates are generally higher in developed countries [1]. However, age is always the most important determinant of PCa [2]. Clinically, the mortality rate among the elderly is significantly increased when compared with other age groups [3]. Indeed, access to good medical care—including screening, diagnosis, and treatment options—is a major factor resulting in lower mortality. Therefore, efforts to reduce mortality are the main goals of the current study. We aim to enable the early detection of PCa through the use of a novel screening biosensor, and we believe that such biosensors could help to achieve new milestones in screening models for the early detection of cancers [4,5,6,7,8,9]. In addition, precise diagnostic and treatment decisions depend on the accuracy of the obtained test results. Differential diagnosis through molecular genetic analysis greatly helps clinicians make informed and rapid decisions about possible treatment options and effectively track the progression of tumors after surgical treatment. These are all effective means to reduce PCa-related mortality.

Clinically, the diagnosis of PCa poses challenges. One major issue is that PCa does not cause noticeable symptoms until it has progressed significantly. Additionally, symptoms such as weak urine stream, pain during ejaculation or urination, and changes in erectile function may also be attributed to benign prostatic hyperplasia (BPH) [10,11] or other non-cancerous conditions, potentially leading to a delay in diagnosis. At present, clinicians mainly rely on screening methods based on prostate-specific antigen (PSA) testing to predict PCa [12]. However, PSA levels may be elevated in not only PCa cases, but also non-cancerous cases, and false positive cases may lead to unnecessary biopsies or treatments. Conversely, some cases of aggressive PCa may not exhibit significantly high PSA levels, which can lead to the possibility of misdiagnosis [13,14]. Therefore, in order to avoid false results, patients may undergo invasive and expensive examinations, such as magnetic resonance imaging (mpMRI), computed tomography (CT) scans, or ultrasound, ultimately leading to unnecessary burden and a waste of medical resources [15]. In this study, we tried to non-invasively collect exosomal miRNA as PCa biomarkers from urine [16], aiming to develop a highly sensitive microRNA biosensor. Based on the detection of PCa-related molecules, the accuracy of PCa diagnosis may be significantly improved. As microRNA biosensors can define the presence of tumors at the genetic level and track the progression of tumors, they are expected to effectively make up for the shortcomings of traditional PSA testing, improve PCa screening guidelines, and address the challenges associated with treatment strategies.

It is an undoubted fact that the analysis of microRNA (miR)—a type of small non-coding RNA that regulates gene expression—has become a clinical trend in cancer diagnosis and treatment strategies [17,18,19,20]. Dysregulation of miRNA-21 in tumor progression has been observed in previous reports focused on kidney disease, cervical cancer, and PCa, among other conditions [21,22,23,24,25,26,27,28]. Accordingly, a homemade probe of an miR-21 nucleotide sequence was applied to a biosensor that we previously developed—namely, a screen-printed carbon electrodes (SPCEs) biosensor—and further optimized via coating with silver nanoparticles [29], which we call the “urinary miR-21 biosensor”. As microRNA in urine is well protected by the bilayer lipid structure of exosomes, thus making it extremely stable and not easily cleavable by external environmental factors, it is considered a very stable and large-volume source for collection from testing specimens [30,31].

In order to verify the detection accuracy and stability of the urine miR-21 biosensor, traditional quantitative reverse transcription polymerase chain reaction (qRT-PCR) technology was used to verify the data consistency. Our results completely confirmed the high sensitivity of the sensor. The analysis of urinary miR-21 expression levels before or after surgery indicated significant differences among the PCa, BPH, and control groups. In addition, all results verified the advantages of the developed biosensor in terms of its high sensitivity and specificity, giving us more confidence that it could be used as a potential adjunctive screening tool for PCa diagnosis in the future.

## 2. Materials and Methods

### 2.1. Reagents and Chemicals

The following components were used for the modification of the SPCE carbon surface. First, carboxymethyl dextran sodium salt (CMD) was applied to the carbon surface, which reacted in order to form COOH functional groups. The residual CMD was then washed using phosphate-buffered saline (PBS). In order to produce a stable chemical structure of acyl groups on the carbon surface through reaction with COOH, 8 mg/mL 1-ethyl-3-(3-dimethylaminopropyl) carbodiimide (EDC) combined with 22 mg/mL N-hydroxysulfosuccinimide (NHS) solution was then mixed with 0.1 M MES buffer at pH 4.7, followed by NeutrAvidin solution, and dripped onto the surface to replace the base for further biotinylated nucleotide probe binding. The functional group –NH2 has been shown to exhibit excellent binding ability with silver nanoparticles (Ag, Ps). Therefore, 10 nm of AgNPs (Sigma-Aldrich, St. Louis, MO, USA) was used to enhance the electrochemical sensitivity of the sensing biochemical assays.

### 2.2. Electrochemical Measuring

The apparatus of a Metrohm AutoLab PGSTAT204 electrochemical workstation (Herisau, Switzerland) was used to measure cyclic voltammetry (CV), and data were analyzed using NOVA1.11. (Herisau, Switzerland). Three of the electrodes were platinum counter-electrodes, a saturated calomel electrode was used as a reference electrode, and the working electrode was a surface-modified SPCE purchased from Zensor R&D (Taichung, Taiwan). In this study, two electrolyte formulations were applied for all tests. First, 0.1 M KCl and 5 mM ferricyanide were used for CV to analyze the surface resistance. Second, a 0.1 M citric acid and 0.2 M sodium phosphate solution was applied for square-wave voltammetry (SWV) measurements with the following settings: −0.5 V deposition potential, 30 Hz frequency, and 0.15 V/s scan rate. Data analysis was performed using Origin 9.0 software (Northampton, MA, USA), and IBM SPSS Statistics 23.

### 2.3. The Oligonucleotide Capturing and Detector Probes Design

**Figure 1:**
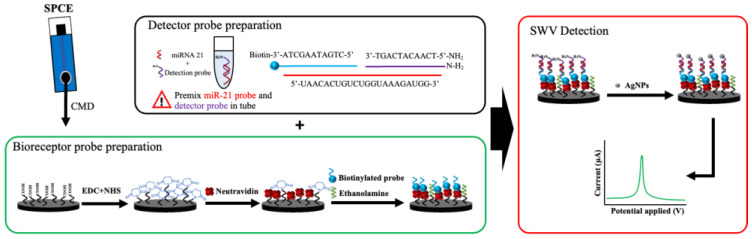
Schematic diagram of the miR-21 biosensor manufacturing process. The silver nanoparticles (AgNPs) were applied to the NeutrAvidin and biotinylated-modified probe on a screen-printed carbon electrode (SPCE)-based sensor strip in order to increase the sensitivity of exosomal microRNA detection.

In this study, the self-designed probes (receptor probe and detector probe), which were made by Genomics (Taipei, Taiwan), were applied to the urine miR-21 biosensor. The probes were mainly designed based on the miRbase sequence database miR-21 sequence, then divided into two parts of the sequence with further modification. The biotinylated oligonucleotide capturing probe was used to link onto the SPCE surface, denoting the receptor probe (5′-CTGATAAGCTA-3′-biotin). The remaining part of the sequence, modified with an amino group (N-H2) on the 5′ end of the oligonucleotide sequence—denoted as the detector probe (H-S-5′-TCAACATCAGT’)—was premixed with the specimens, which was either an artificial mimic targeting the miR-21 sequence (5′-UAACACUGUCUGGUAAAGAUGG-3′) for experimental testing or the urinary miR-21 sequence derived from the clinical patients. Once the amino groups interacted with the silver nanoparticles, the signal was produced via SWV (Figure 1).

### 2.4. Modification of SPCE 

In order to link the receptor probes onto the surface of the SPCE, modification of the SPCE was performed, which was based on our previous report with further optimization [29,32]. First, the surface of the carbon electrode was modified by adding DEPC; then, the carbon electrode was shaken overnight to instill it with 30 μL of 50 mg/mL carboxymethyl dextran sodium salt (CMD) on the surface for 16 h to form carboxylic (COOH) functional groups on the surface. Afterward, 30 μL of EDC-NHS solution (configured with 8 mg/mL EDC and 22 mg/mL NHS mixed with 0.1 M MES buffer at pH 4.7) was instilled on the surface of the carbon electrode and reacted at room temperature for 15 min, after which the leading streptavidin (BioVision Inc. Mountain View, CA, USA) was conjugated on the surface and 5 μM the biotinylated mimicked sequence probe was added to form a functional surface, acting as the “receptor”. Then, 1 M ethanolamine was used to block the remaining activated sites. All experimental solutions were configured using 0.1% DEPC-treated water to inactivate RNase and ensure the stability of reactions [33]. In the current study, silver nanoparticles (AgNPs) were further applied to the NeutrAvidin and biotinylated-modified probe on the screen-printed carbon electrode (SPCE)-based sensor strip in order to increase the sensitivity of exosome microRNA detection.

### 2.5. Urine Samples Collected from Clinical Patients

A total of 90 clinical samples were obtained from the Department of Urology, Chang Gung Memorial Hospital, Linkou, Taiwan. As our aim was to develop a biosensor for PCa, cases that did not meet the criteria of this study—such as patients with other cancers, serious diseases, comorbidities, and so on—were strictly excluded from this study. Therefore, a total of 38 clinical urine samples were obtained from 3 patients diagnosed with BPH; 10 patients diagnosed with PCa with different clinical status at 3 time points (before surgery, 1 month after surgery, and 3 months after surgery); and 5 healthy subjects. The study was conducted under the approval of the Human Subjects Research Ethics Committee/Institutional Review Board (IRB: 202300229B0) of Lin Kou Chang Gung Memorial Hospital (CGMH), and all patients filled out informed consent and personal information statements.

### 2.6. Urinary microRNA Isolation and Measurement from Clinical Samples

To obtain urinary exosomal miR-21 molecules, Norgen’s Urine MicroRNA Purification kit (#29000, Norgen Biotek, Ontario, ON, Canada) was used for the extraction of total RNAs [29], of which those with molecular sizes of less than 200 nt were collected and used. Based on the operating manual, in brief, 1 mL/sample of urine was lysed and centrifuged at 8000 rpm for 5 min, then washed twice and centrifuged at 14,000 rpm. Finally, miRNA was eluted using 50 μL of elution buffer, and the quantity of extracted RNAs was measured using a Multiskan GO spectrophotometer (Thermo Fisher Scientific, Inc., Cleveland, OH, USA). The final concentration yield was around 30 ng/10 μL. Finally, the extracts were stored at −80 °C until use.

### 2.7. miR-21 Quantitative Measurement in Urine via Quantitative Reverse Transcription PCR (qRT-PCR)

To demonstrate the accuracy of the data produced with the proposed miR-21 biosensor, triplicate qRT-PCR examinations were performed to evaluate the expression level of miR-21 in all clinical samples, as well as in samples from healthy subjects (whom were used as a control) for further comparison. First, 10 ng of testing RNA was converted into cDNA using a TaqMan MicroRNA Reverse Transcription kit (Thermo Fisher Scientific, Inc.) [34]. The expression of miR-21 was then quantified through qRT-PCR using a TaqMan MicroRNA Assay kit (Thermo Fisher Scientific, Inc.) and an Applied Biosystems Veriti Thermal Cycler (Thermo Fisher Scientific, Inc.). The level in the 5 healthy control subjects was normalized to 1 and used as a baseline for further comparison.

### 2.8. Statistical Method

In order to further analyze the clinical data of PCa, BPH, and healthy subjects and the differences between the three groups, graphical analysis was conducted using Origin 9.0 software, while IBM SPSS was used for statistical analysis. A *p*-value of less than 0.05 was considered as significant.

## 3. Results 

We had successfully developed miR-141 and miR-451 biosensors in previous studies [33]. However, blurry or weak signals may occur when the samples are unpurified or present low expression levels of target microRNAs. From the current results, it is clear that we made great improvements, in terms of these problems, through the use of silver nanoparticles bonded to the double-stranded detector sequences that successfully hybridized to the sample sequences, as well as the use of receptor probes that release clear signals [35]. This optimization significantly enhanced the signals when using a much lower concentration (i.e., samples with 1 fM). The data were further verified through the use of different electrochemical detection methods, such as cyclic voltammetry (CV). 

### 3.1. Analysis of Electrode Surface Modification

**Figure 2:**
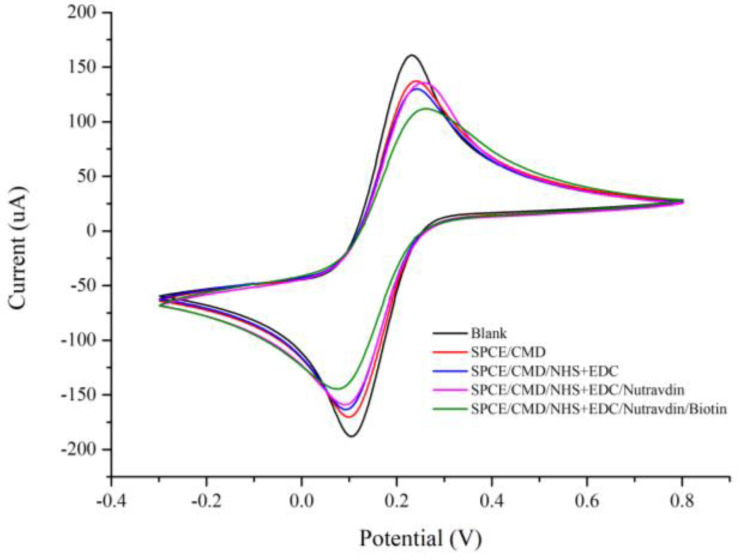
Screen-printed electrode (SPCE) surface modification cyclic voltammetry (CV) results. First, unmodified screen-printed electrodes (shown in blank) were activated with CMD (SPCE/CMD, shown in orange) and then reacted with EDC+NHS solution (SPCE/CMD/EDC+NHS, shown in blue), NeutrAvidin solution (SPCE/CMD/EDC+NHS/NeutrAvidin, shown in pink), and biotin probe (SPCE/CMD/EDC+NHS/NeutrAvidin/Biotin, shown in green).

First, CV measurements were performed to measure the signals derived from the modified surface of the commercial SPCE. As long as the biotinylated probes had successfully captured the detection probes that already hybridized with partial sequences of the targeting miRNA sample, then an appropriate CV scan could be performed, with a rate of 0.1 V/s and a CV range of −0.3 to 0.8 V, under multiple chemical retouching steps (Figure 2), as follows: (1) unretouched screen-printed electrodes (blank line); (2) CMD-activated electrode surface (SPCE+CMD, red line); (3) reaction with NHS/EDC solution (SPCE+CMD+NHS/EDC; blue line); (4) reaction with NeutrAvidin solution (SPCE+CMD+NHS/EDC+NeutrAvidin; pink line); and (5) reaction with biotin probe (SPCE+CMD+NHS/EDC+NeutrAvidin+Biotin; green line) (Figure 2). 

The gradual decrease in current density observed in the CV test confirmed that the SPCE electrode surface had been chemically modified at the different stages, each having a certain impact on the surface resistance of the SPCE, thus further verifying the success of each modification. It can be observed from the CV graph (Figure 2) that the reduction and oxidation peaks were at 0.1 V and 0.3 V for the SPCEs, and the second highest current density value was obtained at the reduction peak after activation of the electrode surface by CMD. The current density at the reduction peak tended to decrease gradually after the subsequent chemical modification steps, and the lowest reduction potential current density was obtained after the biotin probe modification. This confirms that the receptor probe successfully bound to the surface of the SPCE, resulting in higher resistance and lower current density values on the electrode surface.

### 3.2. Optimization of Experimental Parameters

#### 3.2.1. pH Measurement and Testing 

**Figure 3:**
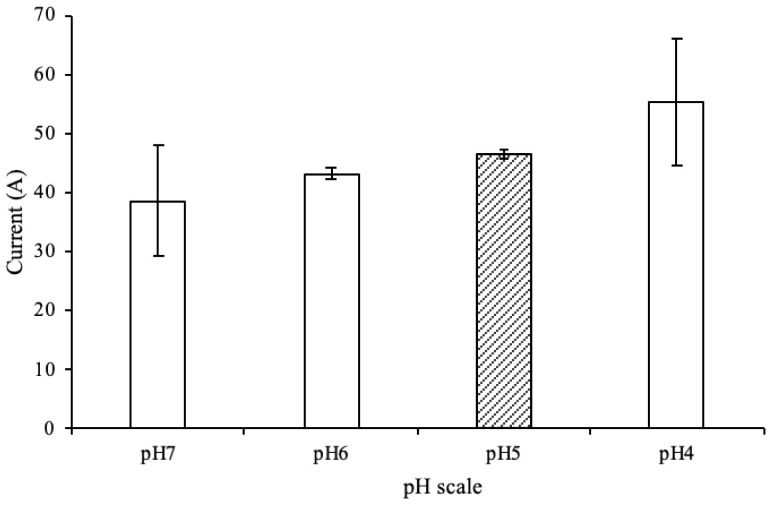
In the interaction of a 1 μM miR-21 sample with the receptor probe on the SPCE, the working electrode responded with different current values at a pH ranging from 4 to 7.

The results indicated that when the pH value decreased, the current signal presented a gradually increasing trend. The region characterized by a pH ranging from 5 to 6 showed the highest stability and acceptability. Therefore, pH 5 was selected for subsequent detection (Figure 3).

#### 3.2.2. Triplicate Stability and Reactivity Experiments of Urinary miR-21 Biosensor

**Figure 4:**
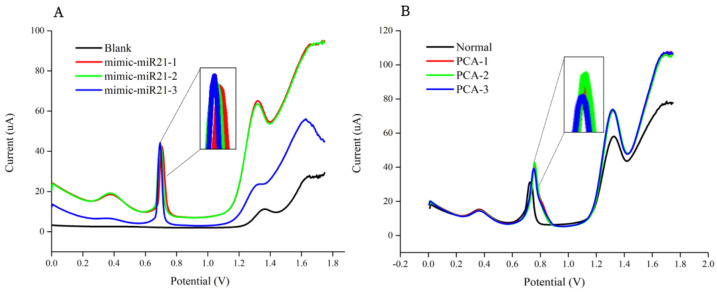
Triplicate stability and reactivity experiments of the urinary miR-21 biosensor were performed via square-wave voltammetry (SWV) using (**A**) a mimic miR-21 oligonucleotide sequence; and (**B**) one of the clinical PCa samples.

In order to obtain accurate clinical diagnosis results, the self-designed probes underwent a two-step hybridization process to accurately hybridize with the target miR-21 sequences, including a mimic miR-21 sequence (Figure 4A) and those from clinical patient samples (Figure 4B). This reaction allowed the sensor to convert the hybridization of the receptor probe to the sample microRNA into a signal, which allows for measurement of the expression levels of the target miR-21.

First, 1 μM mimic oligonucleotide of the miR-21 sequence, which had been reacted with the detector probe, was captured by the SPCE using a modified receptor probe, while 10 μM silver nanoparticles were added as a medium for signal amplification [35]. SWV measurements indicated that, while the current signal was close to 45 μA at potential = 0.7 V, the results obtained with both mimic sequence and clinical samples were consistent, and the receptor probe successfully captured the double-stranded sequence of the target miR-21 sequence hybridized with the detector probe (Figure 4). 

#### 3.2.3. Sensitivity Testing of Urinary miR-21 Biosensor 

**Figure 5:**
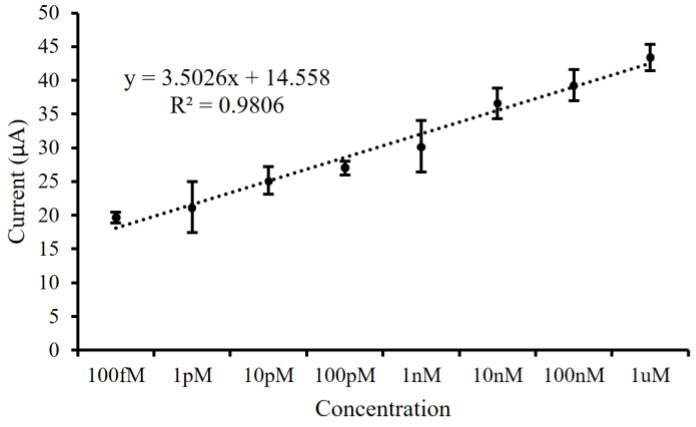
To analyze the sensitivity of the urinary miR-21 biosensor, mimic miR-21 sequences with different concentrations were tested. The square-wave voltammetry current values resulted in a linear relationship with R^2^ = 0.9806.

To test the sensitivity of the urinary miR-21 biosensor, eight different concentrations ranging from 1 μM to 100 fM (i.e., 1 μM, 100 nM, 10 nM, 1 nM, 100 pM, 10 pM, 1 pM, and 100 fM) of mimic miR-21 oligonucleotide sequences were reacted on the sensor. The eight corresponding electrodes were then subjected to SWV measurements. A linear relationship between the current values and individual concentrations was observed after plotting the obtained current values (R^2^ = 0.9806), with the lowest detected concentration value being about 100 fM and the best stability being observed at 1 μM, confirming the high sensitivity of the biosensor (Figure 5).

#### 3.2.4. Selectivity Testing of the Urinary miR-21 Biosensor

**Figure 6:**
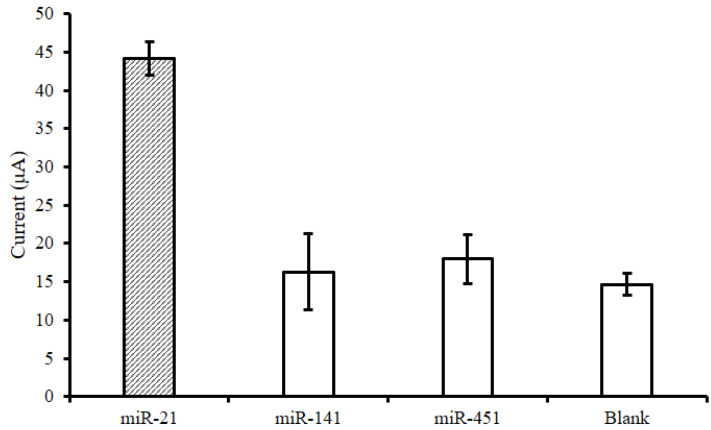
Selectivity analysis of the urinary miR-21 biosensor. The designed receptor probe only targeted the samples with the miR-21 sequence, presenting the highest current signal value of about 45 μA; for the other miRNA sequences, the current values were all lower than 20 μA.

To test the sensitivity of the urinary miR-21 biosensor, 1 μM miR-21, miR-451, and miR-141 were detected, and the results were compared with the signal values of electrodes that had not been reacted with miRNA (Figure 6). When using the urinary miR-21 biosensor, only the miR-21 sequence produced a high current signal value at about 45 μA; meanwhile, for the other sequences of microRNAs, the current signal values were lower than 20 μA. Thus, the extremely high selectivity of the sensor, in terms of its ability to efficiently capture specific target miRNA molecules, was confirmed (Figure 6). 

### 3.3. Validating the Performance of the Urinary miR-21 Biosensor Through Traditional Technology Using Clinical Patient Samples

The urinary miR-21 biosensor was utilized to detect urine samples obtained from PCa patients, BPH patients, and healthy subjects via SWV measurements. All of the samples were then further used to validate the performance of the proposed approach with respect to the traditional qRT-PCR approach. Indeed, both methods presented consistent results. 

#### 3.3.1. Comparison of Results from Clinical Samples Using Urinary miR-21 Biosensors and qRT-PCR

**Figure 7:**
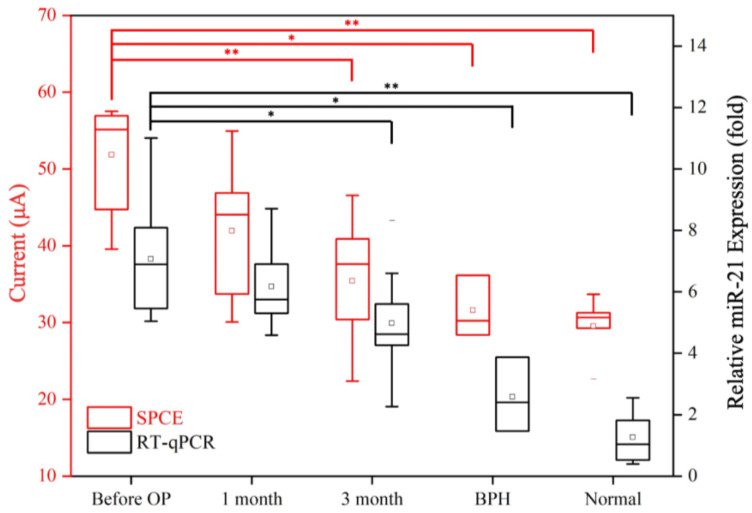
Analysis of 38 clinical urine specimens was performed using the urinary miR-21 biosensor SWV values (red) and qRT-PCR (black) in order to compare the expression levels of miR-21 in PCa (at three time points: before surgery, 1 month after surgery, and 3 months after surgery), BPH, and healthy subjects. * *p*-value < 0.05, ** *p*-value < 0.001.

First, after a triplicate testing analysis for each subject using the biosensor, it was noted that the current signal values of PCa patients were all greater than 45 μA, with some even reaching 60 μA; meanwhile, the current signal values of BPH cases were around 30 μA, and those of healthy subjects were lower than 20 μA. The obvious differences in current values indicated that the proposed urinary miR-21 biosensor is rather stable and reliable, enabling the miR-21 in human urine to be effectively detected and significantly differentiating PCa cases from BPH and healthy cases. Second, considering the results derived from this biosensor, it was verified that the miR-21 molecule is relatively highly expressed in the urine of PCa patients. Importantly, the differences in current values (ranging from 45 μA to above 60 μA) may be correlated with the clinical status or tumor stage, potentially allowing for the estimation of the extent of the cancer (Figure 7).

Furthermore, the urine samples collected at the three time points (before surgery, 1 month after surgery, and 3 months after surgery) from the 10 PCa patients were analyzed (Figure 7). The comparison between PCa cases and healthy subjects, as well as that between PCa cases before and at 3 months after surgery, both indicated highly significant differences (*p*-values < 0.001); furthermore, when compared with BPH, the *p*-value was 0.004. Highly similar results were obtained with the traditional qRT-PCR approach, validating the biosensor SWV values (with 3 months after surgery *p*-value = 0.008, BPH *p* = 0.002, and normal *p* < 0.001). 

#### 3.3.2. Further Confirmation via Analysis of the Level of PSA for the Clinical Cases

**Figure 8:**
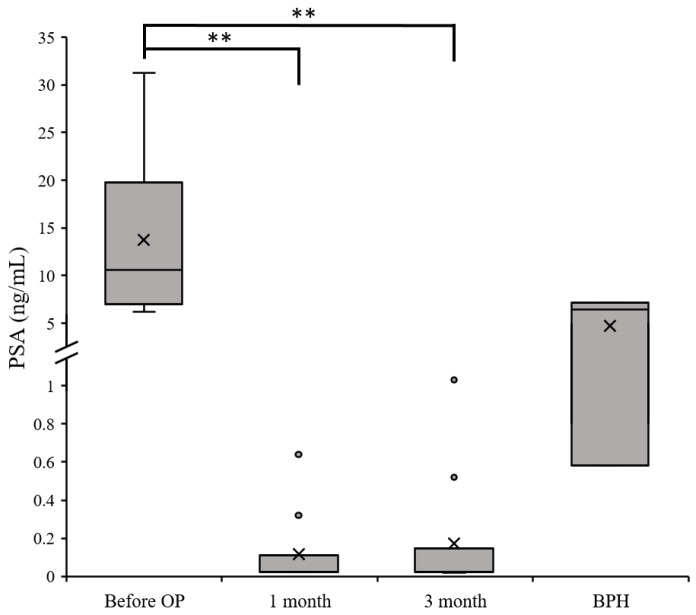
Pre-operative (Before OP) PSA test results for 10 patients with PCa compared with post-operative (1 month and 3 month) results, and those for 3 patients with BPH. High significance was observed in PCa patients before and after OP samples, but no significance was found in benign prostatic hyperplasia cases. ** *p*-value < 0.001.

The blood PSA test results collected from the 10 PCa patients (samples collected at three time points) and 3 BPH patients in this experiment were compared. The results revealed that there were extremely significant differences in the PSA values of PCa patients before surgery, 1 month after surgery, and 3 months after surgery (all *p* < 0.001); however, there were no significant differences in PSA levels between PCa and BPH patients. We conducted a further analysis of individual cases and found that a small number of PCa patients had low pre-operative PSA levels, being quite close to those presented by BPH patients. Such ambiguous PSA data can easily lead to misjudgment by clinicians. This also explains why PSA test results often need to be combined with other clinical examinations to obtain a correct diagnosis (Figure 8).

## 4. Discussion

### 4.1. Evidence from Clinical Samples Shows That Detection of Urinary miR-21 Molecules Is More Sensitive than Blood PSA

Although the conventional blood PSA test results showed that the PSA levels of PCa patients decreased significantly at 3 months after surgery (*p* < 0.001; Figure 8), when compared with BPH patients, there were still a few PCa patients with relatively low PSA values or BPH patients with relatively high PSA levels; as such, it may be difficult to accurately determine the diagnosis of PCa when using PSA levels alone (Figure 8). Meanwhile, when the test samples were analyzed for urine miR-21—either examined using biosensors or conventional qRT-PCR—the results of both methods clearly indicated the presence of miR-21 in the urine of PCa patients. In this study, we proved that a high expression of urinary miR-21 was simultaneously verified with a clinical diagnosis of PCa (Figure 7). From the results of the traditional method (i.e., qRT-PCR), lower expression of urinary miR-21 was not only observed in the specimens at 3 months after surgery, but it was also significantly lower in BPH patients (*p* < 0.05); moreover, the *p*-values were more significant when measured with biosensors (*p* < 0.001 at three months post-operatively and *p* < 0.004 for BPH).

Therefore, we can draw an important conclusion: assessing the expression level of miR-21 in urine may be more accurate and sensitive than detecting PSA in blood. In fact, compared with various current clinical examinations, detecting miR-21 in urine using existing biosensors can be more efficient, can reduce clinical costs, and is a non-invasive examination method. More importantly, the differences between PCa, BPH, and healthy subjects can be easily and quickly distinguished.

### 4.2. Validation of the Effectiveness of the Silver Nanoparticle Platform in Detecting Micromolecules

The most difficult challenges faced when constructing an miRNA biosensor include how to enhance the signal when using relatively low-concentration samples and how to improve the sensitivity, specificity, and stability of the probe. According to our experimental results, we successfully optimized the proposed urinary miR-21 biosensor by adding silver nanoparticles. In particular, the silver nanoparticles effectively amplified the detection signal, producing a 10-fold signal when compared with our previous platform [29], and the selectivity of current sensor greatly improved a lot by about 416–600 times [32]. Higher selectivity and the ability to accurately capture the target sequence help to obtain more accurate current values, thereby increasing the sensitivity of the analysis. Additionally, regarding the limit of detection (LOD) analysis, the minimum concentration 0.1 pM test sample was observed in the current study (Figure 5). We further compared it with other sensors, such as photoactive material-based sensors (LOD is 3.3 aM), surface plasmon resonance sensors (LOD is 0.045 pM), a surface-enhanced Raman spectroscopy optical sensor (LOD is 0.083 fM), and a fluorescence sensor (LOD is 0.1 fM) [36,37,38,39], although some of them showed a higher resolution of analysis. However, the total miRNA concentration in human urine is usually around 60–121 nM, and for some specific miRNAs, their expression actually depends on the disease state. For example, in PCa cases, we observed a range of 1 pM to 10 nM. Therefore, based on the LOD analysis results, our sensor seems to be the best choice for detecting miRNA in the urine of PCa patients, because using a higher-resolution sensor may actually produce more detection interference values. Furthermore, when it comes to the comparison of analysis time, labor consumption, and material costs, although traditional qRT-PCR is still considered a reliable technique so far, after extracting urine miRNA and then reacting it with the sensor or qRT-PCR, speed differences from minutes (3 to 5 min) to 1–2 h must be considered. Additionally, the labor and material costs required vary widely. Indeed, all evidence indicates that, when detecting either artificially simulated microRNA nucleotide sequences or those from clinical samples, the proposed biosensor has highly stable and selective detection capabilities [29]. In addition, in terms of detection speed, the use of SWV also shortens the detection time when compared with the previous chronoamperometric method. 

### 4.3. Current Approaches for Detection of PCa Urine Exosome miRNAs 

Numerous studies have confirmed that the components of exosomes are easily accessible diagnostic biomarkers with great potential for detecting signs of many pathologies, including cancers. However, at present, the identification and quantification of exosomes and their protein, lipid, and RNA contents still requires complex procedures and even high-cost detection methods, such as the use of mass spectrometry, to identify multiple different proteins and exosomes [40]. Therefore, the assays that are commonly used to detect the expression of miRNA usually rely on quantitative reverse transcription PCR [41,42], or patients are asked to transport a urine sample to the laboratory for gene sequencing testing [40]. Comparatively, through the use of the proposed screen-printed electroporation electrochemical-based detection method, combined with our simple exosome miRNA extraction technology and self-designed probes, sample analysis results can be obtained effectively and quickly. 

## 5. Conclusions

Clinically, the Gleason score can predict whether PCa is aggressive, and there is a good positive correlation between the PSA level and Gleason score. However, the PSA value is affected by many factors (e.g., age, race), and some people may have naturally higher PSA values; furthermore, certain drug treatments, prostate hypertrophy, and prostate infection, among other factors, can also affect the PSA value. For the above reasons, a high PSA value does not necessarily mean that a patient has PCa, nor will all PCa patients present a high PSA value in the blood; therefore, after the examination, the results must be carefully interpreted by a medical professional [13,14].

The novelty of this study is that, through changing the signal amplification source of the nucleic acid sensor to silver nanoparticles, the generated signal was enhanced by at least 10-fold, and the sensitivities improved by 416–600 times compared with previous studies [35], greatly optimizing the proposed urine miR-21 biosensor and ultimately improving the sensitivity, selectivity, and stability of the sensor [29,43]. 

In this study, we further demonstrated that urinary exosomal miR-21 is a fairly reliable biomarker that can be used as a stable capture target for biosensors. Our results provided strong evidence that high expression levels of miR-21 in urine could help in the diagnosis of PCa as well as potentially facilitate prediction of the progression of tumors. Significant differences in urinary miR-21 were detected, effectively distinguishing PCa patients from BPH and healthy subjects (BPH patients, *p* = 0.004, and healthy subjects, *p* < 0.001; Figure 7). Similar clinical findings have been reported in several studies, further supporting the results of our study [44,45].

Regarding our 38 clinical samples, we found that detecting miR-21 in urine using biosensors is more accurate for the differential diagnosis of PCa from BPH or healthy subjects when compared with the traditional approach involving the detection of PSA in blood (Figure 8). In addition, the resolution of biosensors is relatively higher compared with detection via qRT-PCR (Figure 7), and the workflow of the biosensor is much more economical, requiring less labor. Ultimately, the biggest benefit associated with the proposed approach is that taking urine samples is non-invasive and non-harmful, which greatly improves patients’ acceptance of being examined, and that it can be repeated countless times (as long as the testing fee can be provided). Finally, biosensors generate clear signals through electrochemical SWV, which allows for specific results to be quickly obtained, allowing effective medical decisions to be made in real time.

## Data Availability

The data that support the findings of this study are available from the corresponding author upon reasonable request.
